# Successful Use of Intravitreal Bevacizumab and Methotrexate in a Case of Neovascularization of the Iris and Pseudohypopyon Secondary to Recurrent Diffuse Large B-Cell Lymphoma

**DOI:** 10.7759/cureus.22578

**Published:** 2022-02-24

**Authors:** Harris Ahmed, Alanna James, Moises Enghelberg

**Affiliations:** 1 Ophthalmology, Loma Linda University Medical Center, Loma Linda, USA; 2 Retina Service, Loma Linda University Eye Institute, Loma Linda, USA

**Keywords:** ocular oncology and medical retina, ocular tumor, general ophthalmology, ocular metastasis, intravitreal therapy, intravitreal injections, intravitreal bevacizumab, surgical retina, vitreo retina, primary orbital lymphoma

## Abstract

In this case report, we aim to describe a rare case of recurrent diffuse large B-cell lymphoma (DLBCL) reportedly in remission presenting with primary anterior segment findings and use of intravitreal bevacizumab and methotrexate to treat the sequelae. The patient presented with hypopyon and neovascularization of the iris (NVI). Anterior chamber studies including flow cytometry and imaging revealed DLBCL recurrence with central nervous system (CNS) involvement. Over one month, he was treated with one intravitreal injection of bevacizumab, repeat injections of methotrexate, and systemic therapies with the resolution of ocular symptoms but persistent systemic disease. This case highlights the utility of anterior chamber paracentesis in diagnosis and intravitreal bevacizumab and methotrexate in the treatment for anterior segment manifestations of intraocular lymphoma (IOL).

## Introduction

Intraocular lymphoma (IOL) can be primary, which typically originates from the central nervous system (CNS), or secondary from metastasis outside of the CNS. It is a rare condition, typically of B-cell origin, and usually associated with primary CNS non-Hodgkin’s lymphoma. Primary IOL usually presents as vitreoretinal lymphoma. Secondary IOL (SIOL) presents with a higher incidence of anterior uveitis, ocular hypertension, and bilateral disease. The prognosis for IOL is poor, with limited treatment options [1].

SIOL is an important diagnosis on the differential in cases of bilateral acute and severe anterior uveitis that recurs with treatment. The most common primary site in these cases is the skin [2]. Iris and anterior segment involvement have only been noted in a few case reports, and even fewer note the usage of anti-vascular endothelial growth factor (anti-VEGF) therapy.

Intraocular lymphoma can be diagnosed histologically with immunohistochemistry, flow cytometry, and/or polymerase chain reaction analysis and suggested by fluorescein angiography, optical coherence tomography, and fundus autofluorescence [3]. Many cases discuss the method of vitreous tap or biopsy for diagnosis, but few studies utilize anterior chamber paracentesis.

The treatment options for IOL are limited and include intravitreal and systemic chemotherapy with methotrexate or radiotherapy [4,5]. Intravitreal rituximab has been used in refractory cases or as an adjunct to methotrexate [6]. If the CNS is also involved, intrathecal methotrexate and cytarabine may also play a role as can rituximab, cyclophosphamide, doxorubicin, vincristine, and prednisone (R-CHOP). Treatment in these cases requires comanagement with oncology. We describe a unique case of anterior segment findings of SIOL and the management of its sequelae.

## Case presentation

**Figure 1:**
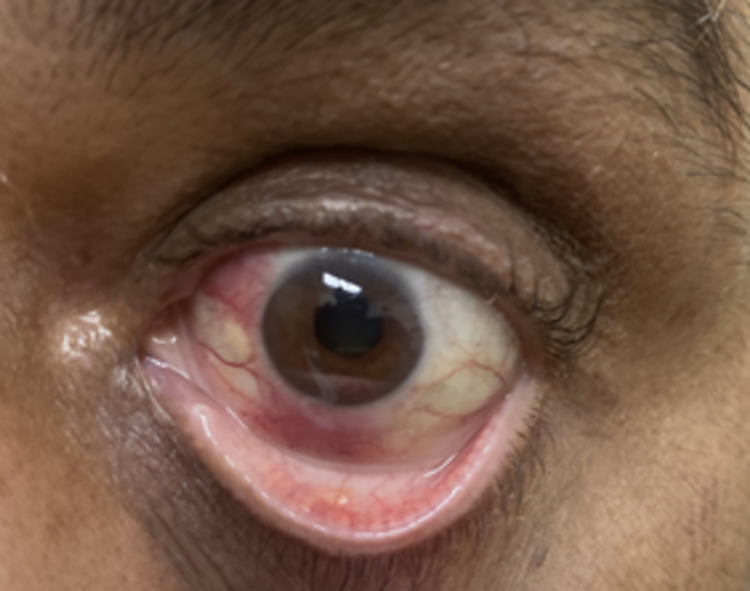
External photograph of the left eye

**Figure 2:**
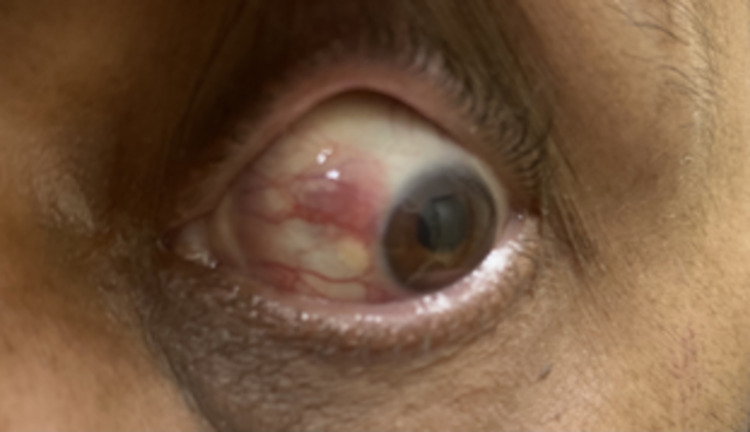
External photograph of the left eye

A 59-year-old Pakistani male with a history of DLBCL in remission presented to the emergency department with a one-month history of left eye redness, decreased vision, and eye pain. Past ocular history was negative. Upon examination, his visual acuity was noted as 20/30 and 20/200 in the right eye and the left eye, respectively. The left eye examination was notable for injection, pink conjunctival lesions at 6 and 10 o’clock, keratic precipitates, a 2.1-mm hypopyon mixed with some blood, 3+ inflammatory cell in the anterior chamber, and neovascularization of the iris (NVI). The left eye displayed mid-peripheral dot and blot hemorrhages. On dilated fundus examination and B-scan, there was no evidence of vitritis (Figures 1, 2).

Past medical history was notable for prior biopsy-proven DLBCL in the maxillary sinus and bilateral testicular involvement. He received six cycles of rituximab, cyclophosphamide, and hydroxydaunorubicin hydrochloride (R-CHOP) and two doses of prophylactic intrathecal methotrexate over the course of several hospitalizations. He also underwent radiotherapy to the right side of his face and bilateral testicles. After a period of remission, he was found to have early relapsed DLBCL based on the punch biopsy of the bilateral calf skin and received three cycles of rituximab, ifosfamide, carboplatin, and etoposide (RICE) treatment and an autologous stem cell transplant. He was considered to be in remission, as his last positron emission tomography (PET)-CT two weeks prior to presentation was without fludeoxyglucose (FDG) uptake in the eye or brain.

**Figure 3:**
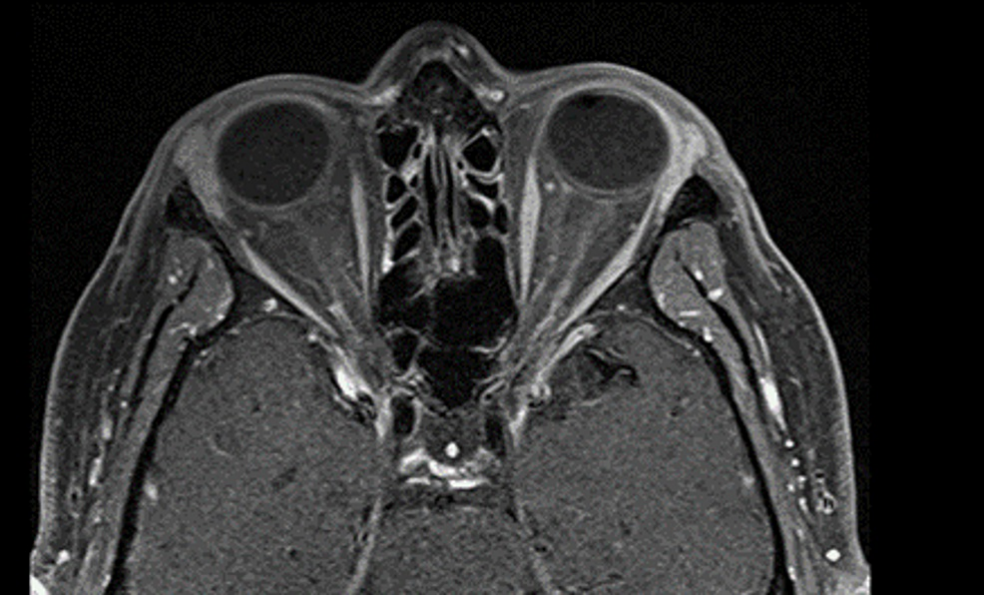
MRI orbits MRI orbits reveal mild inflammatory changes of the left conjunctiva, anterior chamber, anterolateral sclera, left lacrimal gland, and paranasal sinuses.

We performed anterior chamber tap and intravitreal injection with vancomycin and ceftazidime to empirically cover for endogenous endophthalmitis. He was also started on antiviral prophylaxis due to concern for a possible viral etiologic component. MRI orbits showed mild inflammatory changes of the left conjunctiva, anterior chamber, anterolateral sclera, left lacrimal gland, and paranasal sinuses (Figure 3). 

**Figure 4:**
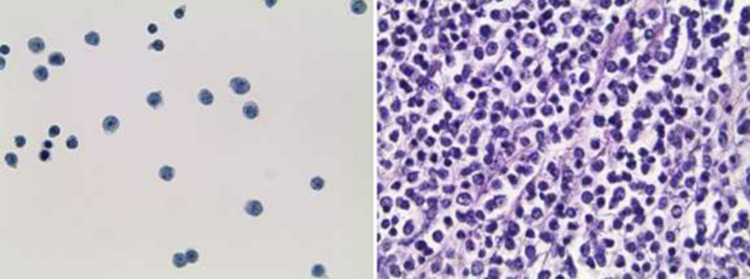
Malignant CD10+ cells present, consistent with known diffuse large B-cell lymphoma (left: thin prep slide, right: cell block)

**Figure 5:**
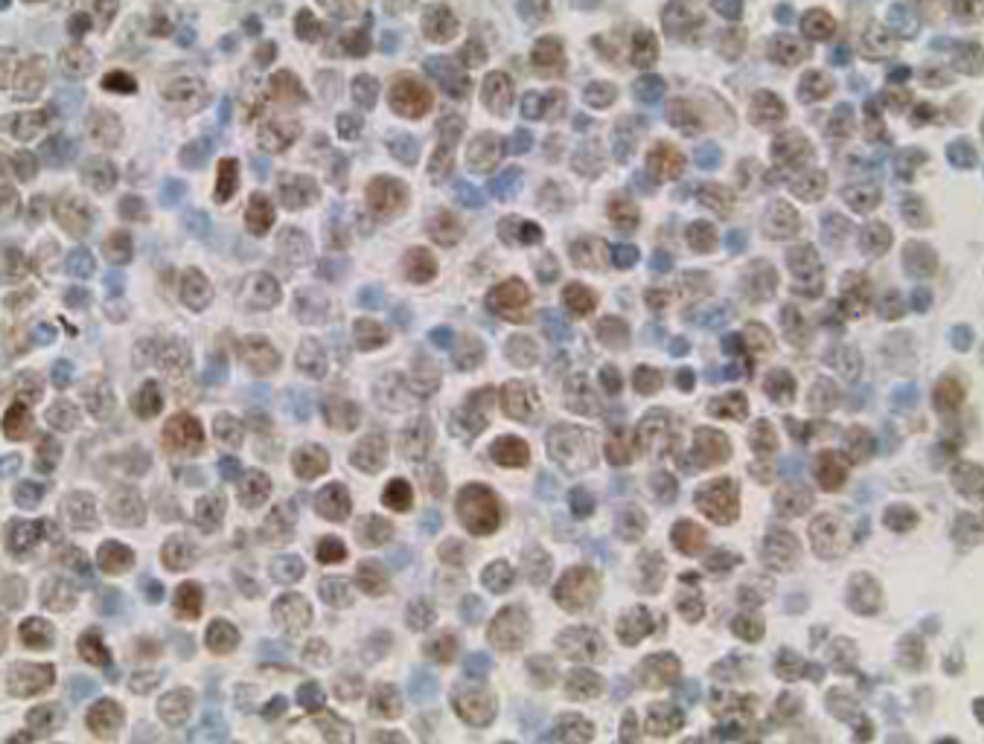
PAX-5 stain with malignant CD10+ cells present consistent with known diffuse large B-cell lymphoma

The anterior chamber tap was sent for flow cytometry, optic culture and sensitivity, and gram stain, which proved negative for infectious etiology and positive for CD10+ mature B-cell lymphoma (Figures 4, 5).

Lumbar puncture for CSF analysis and intrathecal administration of methotrexate were performed. Intravitreal bevacizumab was administered given the iris neovascularization, and the patient was started on prednisolone acetate every two hours in the left eye. The neovascularization resolved two days after the injection of anti-vascular endothelial growth factor. We began twice-weekly intravitreal injections of methotrexate 400 mcg/0.1 mL, planning for one month of this frequency for induction. He received a total of three injections during this admission. During this time, the vision fluctuated from counting fingers at 6 feet to 20/200. By the second injection of methotrexate, the patient’s pain had resolved along with a near resolution of the conjunctival lesions initially noted on admission.

Prior to discharge, the patient was started on ibrutinib 420 mg daily by hematology/oncology as the ocular DLBCL was considered a recurrence of his lymphoma. Lumbar puncture flow cytometry later was found to be positive for B-cell lymphoma as well, indicating CNS involvement.

**Figure 6:**
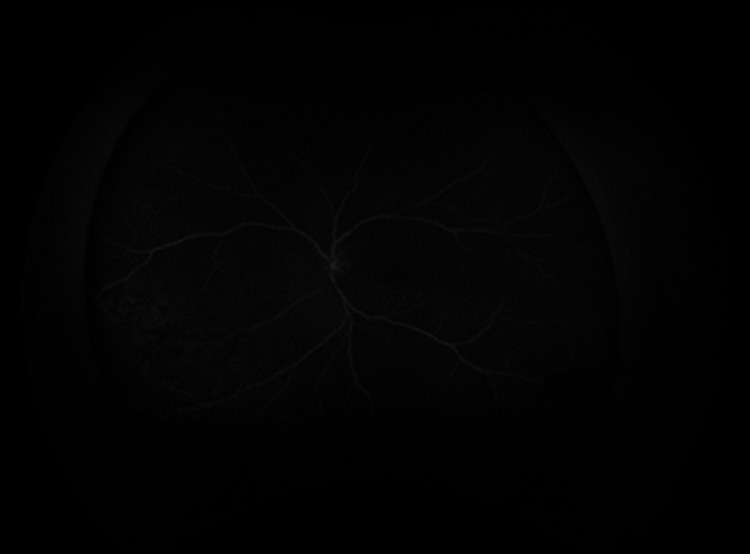
Fluorescein angiography of the left eye demonstrates areas of blockage in the early phases with staining in the mid and late phases

**Figure 7:**
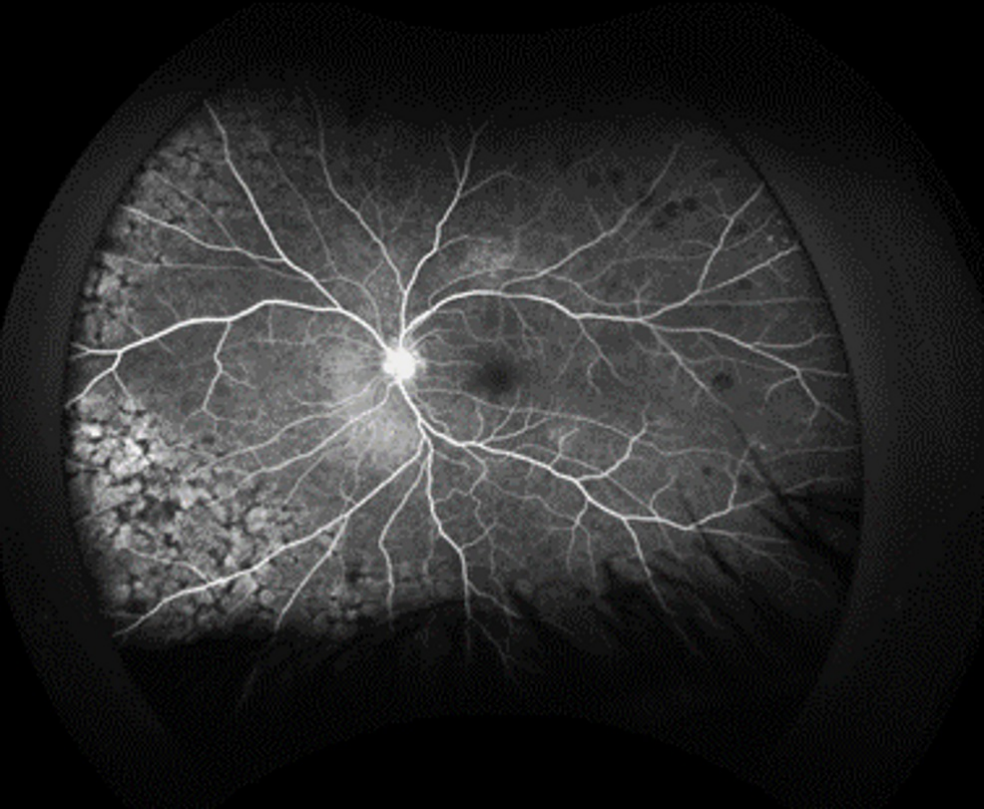
Fluorescein angiography of the left eye demonstrates areas of blockage in the early phases with staining in the mid and late phases

**Figure 8:**
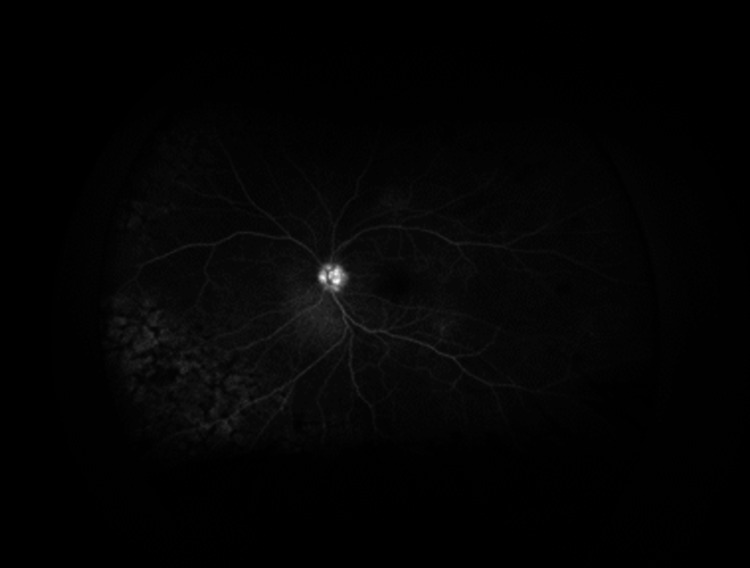
Fluorescein angiography of the left eye demonstrates areas of blockage in the early phases with staining in the mid and late phases

**Figure 9:**
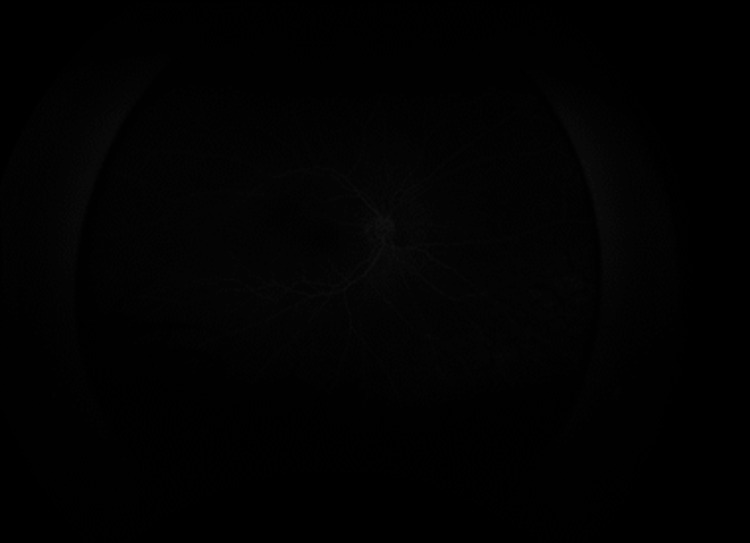
Fluorescein angiography of the right eye reveals areas of staining in early and mid to late phases

**Figure 10:**
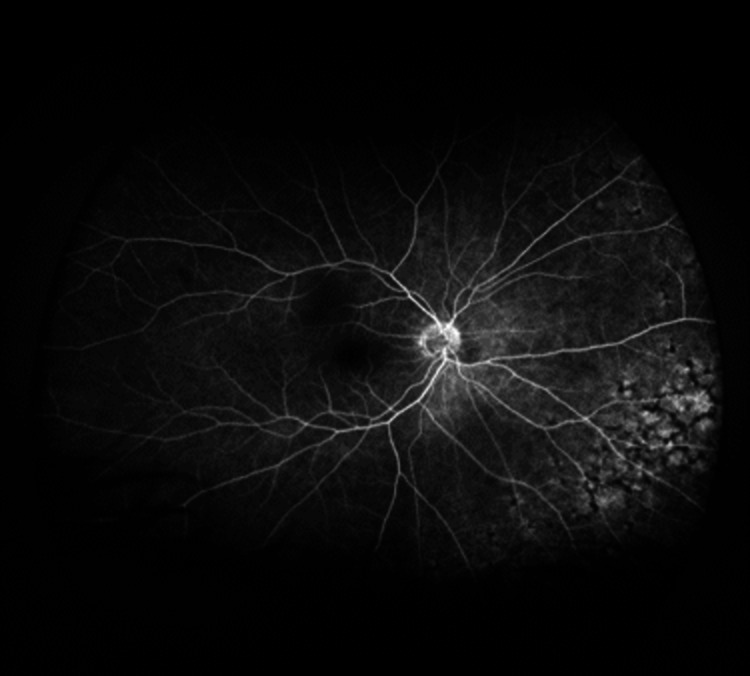
Fluorescein angiography of the right eye reveals areas of staining in early and mid to late phases

**Figure 11:**
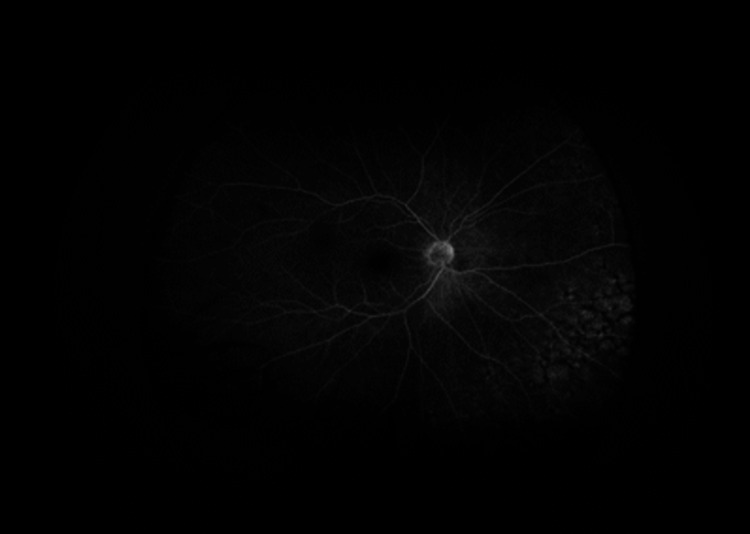
Fluorescein angiography of the right eye reveals areas of staining in early and mid to late phases

As an outpatient, fluorescein angiography (FA) was obtained, and delayed arm to eye time was noted of two minutes (Figures 6-11). Vision remained around 20/200 during this time. Intravitreal injections were stopped due to declining mental status. Over the course of the last year, the patient has had close follow-up with hematology/oncology and has been diagnosed with a relapsed/refractory lymphoma.

## Discussion

Intraocular lymphoma is commonly known as a masquerader and can present as longstanding uveitis refractory to treatment. It can present primarily to an ophthalmologist in cases without any history of lymphoma or cases in remission. Our patient was thought to be in remission with a recent negative PET scan, revealing the importance of a complete eye examination in the diagnosis and treatment of systemic disease. In the setting of previous malignancy, our patient’s presentation was more concerning for secondary intraocular lymphoma, which prompted investigation for a primary tumor with special attention given to the central nervous system.

The gold standard for the diagnosis of IOL is a cytologic specimen from ocular fluid or chorioretinal biopsies [7]. In the past, diagnostic vitrectomy was commonly used to increase the yield. With this method, it is important to obtain an undiluted specimen at a low cut rate [8]. If the sample is not collected correctly, the cells can be damaged for further studies. In patients with a more severe systemic disease, it may prove difficult to proceed with vitrectomy given other patient factors. In our case, the patient tolerated bedside anterior chamber paracentesis well. Finger et al. also found anterior chamber cytology to be effective and less invasive compared to vitrectomy-based biopsy [9].

Our patient had a unique presentation of intraocular lymphoma confirmed by anterior chamber studies and imaging with iris neovascularization (NVI) and delayed arm to eye transit time (ART) on FA. The etiology of NVI in our patient is unclear, but given the pathophysiology of neovascularization, we suspect an ischemic state. Due to the inability to obtain a carotid ultrasound, it is unclear whether this was secondary to carotid occlusion. In the setting of an active malignancy and subsequent hypercoagulability, changes in blood viscosity could be a possible etiology of increased ART [10]. It is possible that the effects of the increased metabolic state of the eye due to the lymphoma resulted in a relatively ischemic state, prompting neovascularization. In our case, the patient presented with florid NVI, which resolved with the administration of intravitreal bevacizumab. The utilization of intravitreal bevacizumab in the setting of intraocular lymphoma is not well studied and has been largely focused on use in the setting of radiation retinopathy and choroidal metastasis. One similar case notes the use of ranibizumab for NVI in secondary IOL from mantle cell lymphoma [11]. The implications of anti-vascular endothelial growth factor (anti-VEGF) therapy on lymphomatous processes are not clear. There is a case report of primary intraocular lymphoma developing despite ranibizumab injections for macular degeneration, indicating that it does not impede the spread of lymphoma in the eye [12]. However, given the chemotherapeutic properties of bevacizumab, the effects could be different but have not been studied in lymphoma.

Interestingly, our patient demonstrated scant vitreoretinal involvement with the majority of his findings limited to the anterior segment. Upon review of the literature, there are few cases of lymphoma presenting predominantly anteriorly. Kitao et al. and Papaliodis et al. documented similar cases with pseudohypopyon as a manifestation of B-cell lymphoma relapse [13,14]. Dorrepaal et al. published a case report of bilateral pseudohypopyon as a manifestation of recurrent vitreoretinal lymphoma [15]. Additionally, this case supports the literature regarding the utilization of intravitreal methotrexate in the setting of lymphoma. Existing literature primarily discussed the utilization of intravitreal methotrexate in the setting of vitreoretinal lymphoma; however, our case sheds light on its use for lymphoma predominantly affecting the anterior segment, which raises the question as to whether methotrexate would have efficacy in similar cases if injected into the anterior chamber or subconjunctivally. As an aside, due to other patient factors, we were only able to complete the induction phase of methotrexate injections, yet there was still noted improvement. Zhou et al. noted that even with a reduced frequency of injections, there was no increase in ocular recurrence [16]. Our case could further emphasize the merit of a shorter regimen in specific patients.

Although our patient did not have known systemic involvement at the time of his ocular relapse, his clinical course has since been complicated by a refractory course with regard to his extraocular lymphoma. Most of the existing literature has focused on DLBCL stage I or stage II. There have been cases in the literature of patients with stage I disease who had no history of lymphoma or associated symptoms outside of ocular symptoms and presented to the ophthalmologist, ultimately leading to a diagnosis of DLBCL. It is notable that there were no signs of recurrence on the recent PET-CT scan despite significant intraocular involvement and later cutaneous involvement noted, which raises the question of the utility of PET-CT in screening for lymphoma recurrence and specifically ocular spread.

## Conclusions

There are few cases in the literature of lymphoma recurring intraocularly; however, it is noted that intraocular involvement is associated with a high risk of relapse of CNS lymphoma and poor prognosis. Thus, intraocular lymphoma remains an important differential diagnosis of refractory uveitis as it is important in the detection and prognosis of systemic disease. In our case with stage IV disease, the patient was treated with intravitreal methotrexate in addition to the reinstitution of systemic chemotherapy. Despite the improvement of some and the resolution of other ocular symptoms, the patient still remains with systemic DLBCL and subsequent complications.

Our case discussed a rare presentation of intraocular lymphoma with anterior segment findings in the setting of a history of systemic DLBCL thought to be in remission. While ocular lymphoma may present in a variety of ways, our patient presented with pseudohypopyon, as well as neovascularization in one eye, without significant posterior involvement. In our patient, the intraocular lymphoma workup ultimately led to a discovery of DLBCL recurrence and subsequent treatment with intravitreal methotrexate and bevacizumab with ocular improvement, along with intrathecal methotrexate and systemic ibrutinib. The unique elements of this case include the ocular presentation leading to the discovery of insidious CNS lymphoma, diagnosis of relapse using anterior chamber paracentesis, utilization of intravitreal bevacizumab for neovascularization secondary to IL, and use of intravitreal methotrexate for anterior segment manifestations of intraocular lymphoma.
